# Antibiotic Resistance Risk with Oral Tetracycline Treatment of Acne Vulgaris

**DOI:** 10.3390/antibiotics11081032

**Published:** 2022-07-30

**Authors:** Madisen A. Swallow, Ryan Fan, Jeffrey M. Cohen, Christopher G. Bunick

**Affiliations:** 1 Yale School of Medicine, New Haven, CT 06510, USA; madisen.swallow@yale.edu (M.A.S.); ryan.fan@yale.edu (R.F.); 2 Department of Dermatology, Yale School of Medicine, New Haven, CT 06510, USA; jeffrey.m.cohen@yale.edu

**Keywords:** antimicrobial resistance, acne vulgaris therapy, tetracycline antibiotic adverse events, dermatologic skin disease, *Cutibacterium acnes*

## Abstract

Almost 1 billion people worldwide have acne, and oral tetracyclines, including doxycycline and minocycline, are effective and frequently prescribed treatments for acne. However, there is growing concern for the development of antibiotic resistance with such widespread utilization by dermatologists. Additionally, tetracyclines are known to have various potential side effects, including gut dysbiosis, gastrointestinal upset, photosensitivity, dizziness, and vertigo. However, in 2018 a novel narrow-spectrum tetracycline, sarecycline, was Food and Drug Administration-approved to treat moderate-to-severe acne vulgaris in patients 9-years-old and above. Sarecycline was designed to target *Cutibacterium acnes*, the pathogenic bacterium in acne vulgaris, which may reduce the risk of resistance. This paper examines the growing concerns of antibiotic resistance due to oral tetracycline usage in the treatment of acne vulgaris, with a focus on the promising third-generation, narrow-spectrum tetracycline, sarecycline.

## 1. Acne Vulgaris

Acne vulgaris is a common skin disease growing in prevalence and is currently the eighth most prevalent disease globally [1,2]. In 2019, acne vulgaris resulted in 3.52 million disability-adjusted life years for patients ages 15–49 years old, and 4.96 million overall [3]. Acne impacts around 85% of people aged 12 to 25 in the United States [4]. However, acne is not just an adolescent condition, as it continues to affect between 40–50% of adults in their 20s and between 20–35% of adults in their 30s [5]. Acne is the leading skin condition resulting in permanent scarring and with significant psychological impact [6]. The reported negative psychosocial effects of acne include a lack of strong friendships, lack of romantic relationships, and failure to engage in school or work [7,8,9]. Additionally, one study revealed that females with significant acne demonstrated suicidal ideation twice as frequently as those with mild acne (25.5% vs. 11.9%), and males with significant acne showed suicidal ideation three times as often as those with mild acne (22.6% vs. 6.3%) [10].

Acne vulgaris is an inflammatory condition of the pilosebaceous unit [11]. Pathogenesis of this disease is multifaceted, involving four inter-connected processes: inflammation, increased sebum production, hyper-keratinization of the follicular infundibulum, and proliferation of *C. acnes* [4]. This condition often coincides with the increased levels of androgens and increased sensitivity of androgen receptors during puberty [11]. Diagnosis is most often clinical, depending on history, symptoms, and clinical examination [12]. The most common characteristic lesions are closed comedones, open comedones, inflammatory papules, pustules, inflamed nodules and inflamed nodulocystic lesions, most typically distributed on the face, neck, back, chest, shoulders, or upper arms [6].

## 2. Oral Antibiotic Treatment: Tetracyclines

First-line treatment for mild to moderate acne include topical agents, while combination therapy and/or systemic therapies are recommended for moderate-to-severe acne [6]. Systemic treatments include oral antibiotics such as tetracyclines, macrolides, and trimethoprim/sulfamethoxazole, which have been determined to be effective and safe in the treatment of moderate-to-severe acne [13]. Head-to-head studies comparing the efficacy of these therapies are lacking, but tetracyclines are generally considered first-line therapy, as macrolides have been associated with increasing antimicrobial resistance and trimethoprim/sulfamethoxazole has a less favorable side effect profile (including gastrointestinal disturbance and allergic skin reactions) [13]. Additionally, tetracyclines are beneficial due to their effective anti-inflammatory and antimicrobial characteristics, and they account for 75% of all oral antibiotics prescribed in dermatology [14]. The most common tetracyclines prescribed for acne are doxycycline and minocycline [14]. In a phase II multicenter trial, modified release 40 mg doxycycline was proven to be statistically significant to the placebo, resulting in a 41.7% reduction in total lesions vs. 35.9% for the placebo [15]. Similarly, a phase III multicenter trial found that patients receiving 1 mg/kg daily extended-release minocycline had a 43.1% reduction in inflamed lesions vs. 31.7% for placebo [16].

## 3. The Risk of Antibiotic Resistance

**Figure 1:**
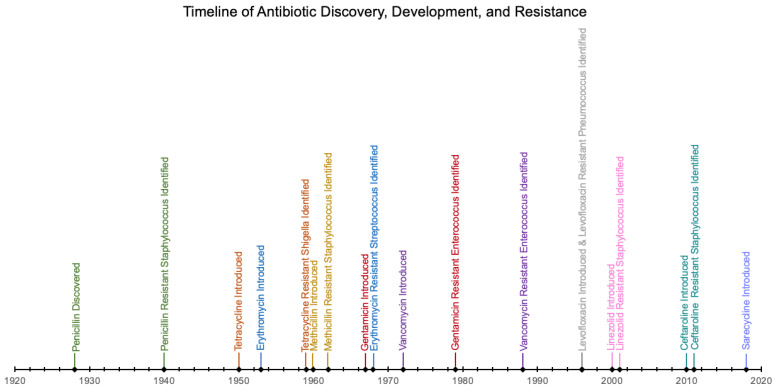
Timeline of antibiotic discovery, development, and resistance. Adapted from Centers for Disease Control and Prevention [21].

A growing serious threat to human, animal, and environmental health worldwide is antimicrobial resistance (Figure 1) [17]. The key influencing factors for antibiotic resistance development include inattentive use of antibiotics and failure to develop novel antibiotics [18]. Due to the increasing rates of antibiotic resistance, the World Health Organization has deemed it a critical global public health issue of this century [19]. Challenges and financial burdens from antibiotic resistant pathogens have been seen on every continent in the world [20]. For example, sediment samples containing bacteria from the Netherlands have demonstrated an increase in antibiotic resistant genes with resistance against tetracyclines, and samples from China identified ten different resistant genes [19]. The World Economic Forum demonstrated that in 2013 in North America, the majority of 99,000 deaths from hospital-acquired infections per year were caused by antibiotic-resistant bacteria and resulted in healthcare costs ranging from USD 21–34 billion [20].

**Figure 2:**
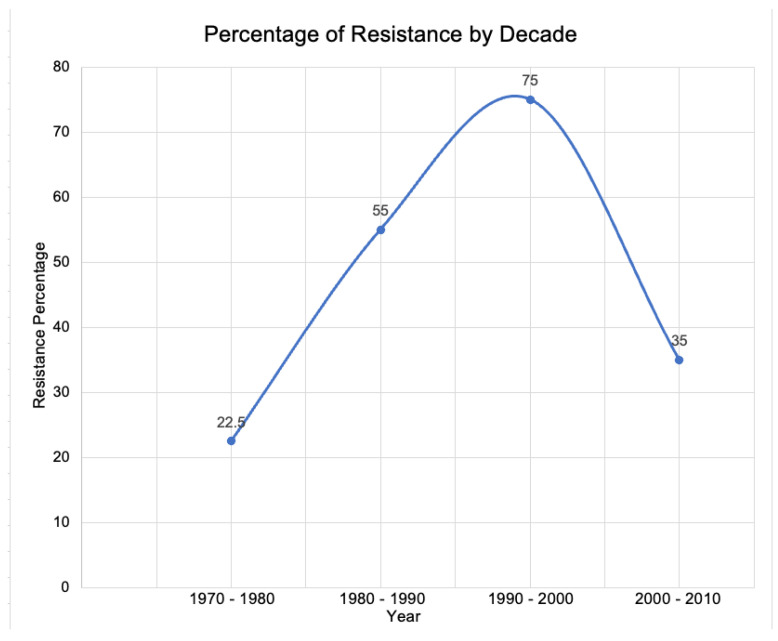
Approximate percentage of antibiotic resistance by decade. Data adapted from Karadag et al. [25].

Topical and oral antibiotic use in acne treatment has been associated with the increasing resistance seen in *C. acnes* [22,23]. A study in 1976 demonstrated that there was no antibiotic resistant *Propionibacterium acnes* in over 1000 patients with acne vulgaris [24]. However, resistance soon started to develop, with resistance rates at approximately 20–25% in the 1970s to 1980s, 50–60% in the 1990s to 2000s, and 75% in the 2000s to 2010s (Figure 2) [25]. However, between 2010 and 2020, levels of resistance fell to 30–40% (Figure 2) [25]. This fall in resistance levels could be attributed to improved understanding of the dangers of antibiotic resistance, and changes in prescribing habits resulting in a decrease in their superfluous usage by clinicians. It is unlikely the decrease is due to the development of new antibiotics targeting *C. acnes* since the one new agent, sarecycline, did not become available in the United States until 2018. This is too late in the decade to make a significant impact in the data. Additionally, differences in various locations are substantial, with resistance between 2010 and 2020 seen at 54.8% in Hong Kong, while only 9% in Australia [25]. Large countries also demonstrate a wide range in reports of resistance, with China ranging from 6.1 to 90.4% and 10.6 to 98% in India [25]. The mechanism of action for tetracyclines include binding bacterial ribosomes in the highly conserved 16S ribosomal RNA target in the 30S ribosomal subunit, resulting in termination of translation by steric interference with the docking amino-acyl-transfer RNA [26,27,28]. Hence, there are various mechanistic reasons for tetracycline resistance including mutations in the ribosomal binding site, increased genetic components with tetracycline-specific resistance genes, or mutations in chromosomes causing an elevated expression of intrinsic resistance mechanisms [29].

Additionally, those taking oral antibiotics for acne treatment typically take the medications for 3–6 months or longer, increasing the opportunity for developing resistance [23]. Studies show that resistance increases with duration of acne, older age, and duration of treatment [30]. It has also been demonstrated that there is a higher level of resistance in patients who were treated with antibiotics for acne before [31]. Additionally, there is significant correlation for resistance between multiple antibiotics, with 5.6% of 36 tested strains of *C. acnes* being resistant to all antibiotics tested in one study [32].

Given the multifactorial pathophysiology of acne, resistance in this context can present in variable ways including decreased response to therapy, no response to therapy, or relapse of disease [33]. It has been previously demonstrated that decreased clinical efficacy can occur from antimicrobial resistance with erythromycin [33]. In addition, antibiotic resistance can result in systemic effects [22]. One study demonstrated that following even a 7-day course of oral antibiotics the gut microbiome can be altered for 2 years afterwards [34]. One retrospective cohort study demonstrated a significant increase in likelihood of developing an upper respiratory infection 1 year following treatment with topical and/or oral antibiotics for more than 6 weeks [35]. Another study demonstrated that receiving oral antibiotics resulted in a more than 3-fold likelihood of reporting pharyngitis 1-year following treatment [36].

## 4. Other Negative Effects of Traditional Tetracycline Usage

In addition to the rise in antibiotic resistance seen with the increase in oral tetracycline usage, there are various other risks associated with long-term use of tetracyclines. Doxycycline use has been associated with gut dysbiosis and increased risk of irritable bowel disease and inflammatory bowel disease [37]. For example, one study demonstrated a hazard ratio of 2.25 for the development of Crohn’s disease after being prescribed doxycycline for acne [38]. It was also noted that there is an associated risk for breast and colon cancer with long-term antibiotic use; however, more data is needed to be conclusive [14]. One recent review discussed the importance of the microbiome and its connection to cancer homeostasis, with clinical data demonstrating that systemic antibiotics can terminate checkpoint efficacy, resulting in a decreased survival [39,40,41,42]. Tetracyclines have also been noted to cause phototoxicity, urticaria, and lupus-like syndrome [43]. These antibiotics can cause tinnitus, pseudotumor cerebri, or vertigo with the latter adverse event leading to restrictions on minocycline use in military aviators [37,44]. Minocycline can cross the blood–brain barrier, potentially explaining the increased risk for dizziness and vertigo [43,45]. Although topical minocycline has a low resistance claim on its label, oral minocycline does not. Finally, candidiasis or vulvovaginal mycotic infections are also known side effects of tetracycline usage [37].

## 5. Benefits of Sarecycline: Reduced Antibiotic Resistance

**Figure 3:**
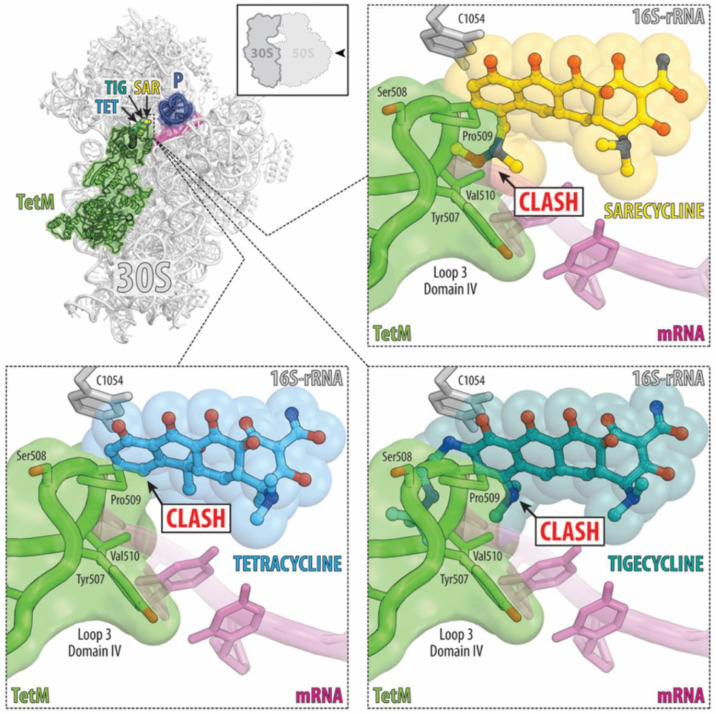
Structural basis for sarecycline’s low propensity for antimicrobial resistance. (**Upper left**) Superposition of the structure of ribosome-bound ribosomal protection protein TetM (green, Protein Data Bank ID code 3J9Y) [54] with the structures of ribosome-bound tetracycline (TET, blue, PDB ID code 4V9A) [55] tigecycline (TIG, teal, PDB Code 4V9B) [54] and sarecycline (SAR, yellow, PDB ID codes 6XQD and 6XQE) [53]. All structures were aligned based on the 16S rRNA. mRNA is colored magenta; P-site tRNA is dark blue. (**Upper right**) Close-up view of the steric clash caused by the C7 moiety of SAR with Pro509 and Val510 of loop 3 of domain IV of TetM, thereby preventing access to the SAR binding site by TetM. (**Lower right**) TIG has an extended C9 moiety in addition to a smaller C7 moiety than SAR, both of which cause steric clashes with Ser508, Pro509, and Val510 of loop 3 of domain IV of TetM. (**Lower left**) TET, in contrast, does not have C7 or C9 moieties, meaning its ability to restrict TetM access to the decoding site of the 30S bacterial ribosome is limited to a minor steric clash with Pro509. Figure courtesy of Christopher Bunick from Batool 2020 [53].

In October of 2018, the U.S. Food and Drug Administration (FDA) approved sarecycline, a third-generation, narrow-spectrum tetracycline-derived antibiotic, as a treatment for moderate-to-severe acne in patients 9-years-old and above [46]. Like other broad-spectrum tetracyclines popularly used for the treatment of acne, sarecycline has antibacterial and anti-inflammatory effects [46,47]. In addition, sarecycline has a less potent effect on various other types of bacteria, especially Gram-negative intestinal microbial flora, resulting in a more specific, targeted treatment for *C. acnes* strains [46,48,49]. Sarecycline has a longer half-life (21–22 h compared to minocycline’s half-life of 16–19 h and doxycycline’s half-life of 16–22 h), resulting in the ability to dose it daily with or without food, which is beneficial because it increases compliance among patients [50,51]. Additionally, when compared with other tetracyclines, sarecycline has exhibited low tendency for antibiotic resistance [43]. It is the only oral antibiotic approved for acne with a low risk of resistance claim in its label [46]. Phase 1, 2 and 3 studies demonstrated the safety and efficacy of sarecycline usage for acne on the face and/or trunk [39,52]. Phase 3 studies demonstrated a 51.8% decrease in inflammatory lesions with sarecycline usage [39]. In vitro studies have also demonstrated less activity against Gram-negative bacteria in the human gut microbiota and reduced blood–brain barrier penetration, which likely aided in the reduction of adverse events seen in clinical trials [39,48,52]. Furthermore, it has been demonstrated from X-ray crystallography that the unique chemical group, the carbon 7 (C7) moiety, on sarecycline is critical for establishing biochemical properties for sarecycline that distinguish it from doxycycline and minocycline. These structural and biochemical differences are directly related to sarecycline’s decreased propensity for inducing antibiotic resistance, as illustrated by sarecycline’s increased ability to cause steric clash with the ribosomal protection protein TetM (Figure 3) [45,53].

Despite the potential benefits of sarecycline, its significantly higher price compared to other tetracyclines such as doxycycline and minocycline, as well as lack of coverage by Medicare part D and other insurance plans, may limit its wider usage. This is a significant problem because lack of access to sarecycline hinders the ability for dermatologists and other medical providers to practice appropriate and necessary antibiotic stewardship [45]. At some point the cost of precision medicine care upfront (narrow-spectrum sarecycline) must be viewed as advantageous compared to the higher overall healthcare costs that may ensue downstream from the complications of broad-spectrum antibiotic use.

## 6. Conclusions and Outlook

Antibiotic resistance continues to be an increasing serious danger to health worldwide. The global burden of resistance in 2019 demonstrated that approximately 4.95 million deaths were associated with antimicrobial resistance [3]. The data revealing a decrease in antibiotic resistance in the 2010–2020 decade (Figure 2) seems at odds with the global resistance burden still observed in 2019. The problem of antibiotic resistance is multifactorial, and blaming it all as a consequence of unconcerned use of antibiotics by physicians does not seem fair or accurate. In the acne vulgaris space there clearly has been a lack of development of novel antibiotics, particularly innovation of targeted, narrow-spectrum antibiotics besides sarecycline that preserve the host microbiome. Advances in antibiotic stewardship in clinical medicine require more than alteration in physician thinking or prescribing habits; in particular, industry needs to innovate and invest more in developing antibiotics that reduce resistance risk and payors (insurance companies) need to more constructively use their policies to facilitate access to new cutting-edge antibiotics with favorable resistance risk even if at a higher price.

*C. acnes* resistance is also important for various other fields beyond dermatology. Resistance to *C. acnes* is a major problem in orthopedics because of joint infections during surgery. It is the most common bacteria to cause complications following shoulder arthroplasty and due to its difficulty in culturing, it may take up to 17 days for a positive result [56]. Additionally, antibiotic resistance goes beyond healthcare, with concerns growing within the livestock industry. In 2018, McDonald’s made a pledge to reduce antibiotics in the beef supply chain and by 2021 many advocacy groups continued to be frustrated as the company failed to reach the created targets for antibiotic use reduction [57]. Additionally, the WHO recently highlighted the continuing inadequacy of innovation of new antibiotics, stating that in the past five years only 12 antibiotics were approved, and 10 are in existing classes with well-known mechanisms of antimicrobial resistance [58]. Unfortunately, it is predicted that by 2050 there will be 10 million deaths per year globally from antimicrobial resistance, which is more than the deaths from COVID-19 in 2020 [59]. However, there is hope on the horizon as the AMR Action Fund plans to invest more than one billion US dollars towards the development of novel antibiotics [59].
